# (−)-Benzyl 2,3-dide­oxy-β-d-*erythro*-hex-2-eno­pyran­oside

**DOI:** 10.1107/S1600536813031140

**Published:** 2013-11-23

**Authors:** Shigeru Ohba, Hayato Okazaki, Yuji Ueda, Kengo Hanaya, Mitsuru Shoji, Takeshi Sugai

**Affiliations:** a Research and Education Center for Natural Sciences, Keio University, Hiyoshi 4-1-1, Kohoku-ku, Yokohama 223-8521, Japan; b Department of Pharmaceutical Science, Keio University, Shibakoen 1-5-30, Minato-ku, Tokyo 105-8512, Japan

## Abstract

In the title compound, C_13_H_16_O_4_, the six-membered ring of the sugar moiety shows a half-chair conformation. In the crystal, mol­ecules are connected *via* O—H⋯O hydrogen bonds, forming columns around twofold screw axes along the *b-*axis direction. There is a disorder of the benz­yloxy group, which has two possible orientations with the phenyl group lying on a common plane [site-occupancy factors = 0.589 (9) and 0.411 (9)].

## Related literature

For the phenolic Ferrier reaction, see: Ferrier & Prasad (1969 ▶); Noshita *et al.* (1995 ▶). For the structure of the related compound α-glycoside, see: Wingert *et al.* (1984 ▶). For the synthesis of β-glycoside, see: Di Bussolo *et al.* (2002 ▶, 2004 ▶). For the enzymatic regioselective acyl­ation of d-glucal, see: Calveras *et al.* (2010 ▶).
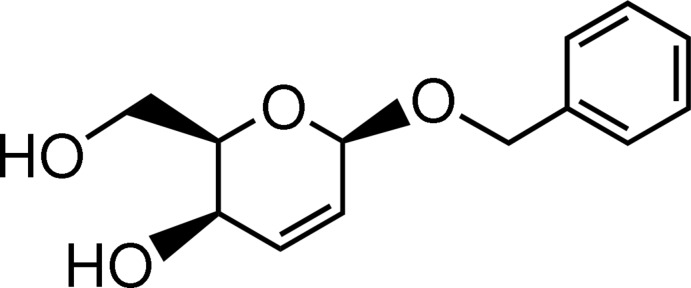


## Experimental

### 

#### Crystal data

C_13_H_16_O_4_
*M* *_r_* = 236.26
Monoclinic, 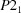

*a* = 19.500 (9) Å
*b* = 5.291 (2) Å
*c* = 6.0809 (15) Å
β = 94.27 (3)°
*V* = 625.7 (4) Å^3^
*Z* = 2
Mo *K*α radiation
μ = 0.09 mm^−1^
*T* = 292 K
0.60 × 0.40 × 0.20 mm

#### Data collection

Rigaku AFC-7R diffractometer
1713 measured reflections
1585 independent reflections
786 reflections with *F* ^2^ > 2σ(*F* ^2^)
*R*_int_ = 0.034
3 standard reflections every 150 reflections intensity decay: 0.8%

#### Refinement

*R*[*F* ^2^ > 2σ(*F* ^2^)] = 0.056
*wR*(*F* ^2^) = 0.255
*S* = 1.04
1579 reflections
193 parameters
33 restraints
H-atom parameters constrained
Δρ_max_ = 0.16 e Å^−3^
Δρ_min_ = −0.17 e Å^−3^

### 

Data collection: *WinAFC Diffractometer Control Software* (Rigaku, 1999 ▶); cell refinement: *WinAFC Diffractometer Control Software* (Rigaku, 1999 ▶); data reduction: *CrystalStructure* (Rigaku, 2010 ▶); program(s) used to solve structure: *SIR92* (Altomare *et al.*, 1993 ▶); program(s) used to refine structure: *SHELXL97* (Sheldrick, 2008 ▶); molecular graphics: *ORTEPII* (Johnson, 1976 ▶); software used to prepare material for publication: *CrystalStructure* (Rigaku, 2010 ▶).

## Supplementary Material

Crystal structure: contains datablock(s) General, I. DOI: 10.1107/S1600536813031140/is5321sup1.cif

Structure factors: contains datablock(s) I. DOI: 10.1107/S1600536813031140/is5321Isup2.hkl

Supplementary material file. DOI: 10.1107/S1600536813031140/is5321Isup3.cdx

Additional supplementary materials:  crystallographic information; 3D view; checkCIF report

## Figures and Tables

**Table 1:** Hydrogen-bond geometry (Å, °)

*D*—H⋯*A*	*D*—H	H⋯*A*	*D*⋯*A*	*D*—H⋯*A*
O2—H2⋯O3^i^	0.82	1.95	2.692 (8)	151
O3—H3⋯O2^ii^	0.82	1.89	2.614 (8)	147

Symmetry codes: (i) 
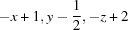
; (ii) 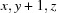.

**Fig. 1.**
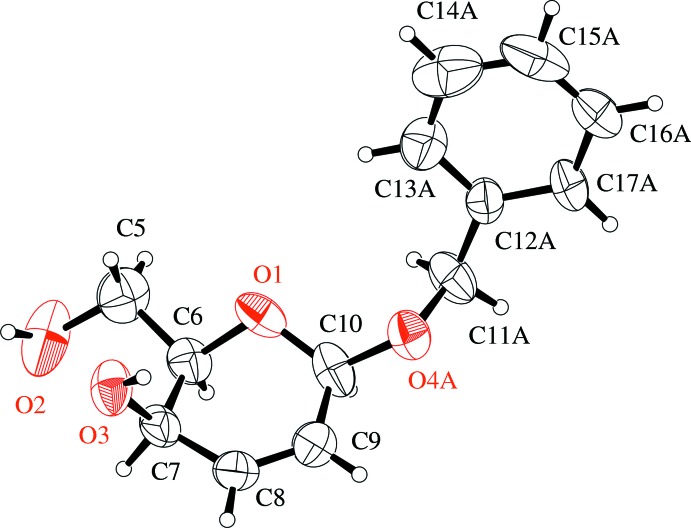
Molecular structure of the title compound with anisotropic displacement parameters drawn at the 30% probability level. The minor part of the disordered benzyloxy group was omitted for clarity.

**Fig. 2.**
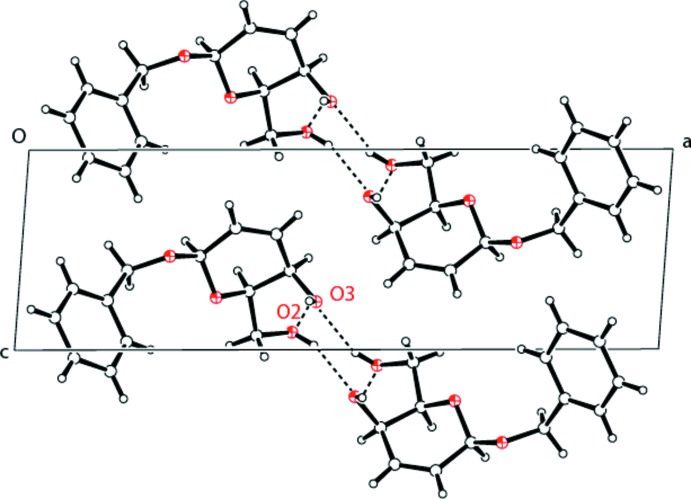
Crystal packing viewed along the b axis with intermolecular O—H···O hydrogen bonds as dashed lines. The minor part of the disordered benzyloxy group was omitted for clarity.

**Fig. 3.**
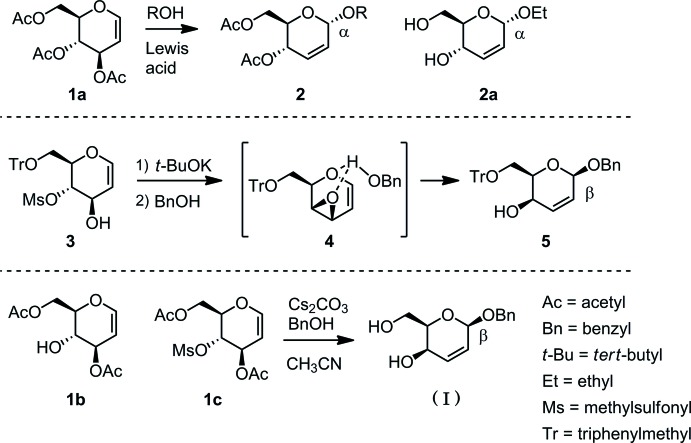
Ferrier reaction and synthesis of the title compound.

## supplementary crystallographic information

### 1.1. Synthesis and crystallization

To a solution of 1c (669 mg, 2.17 mmol) in CH_3_CN (22 ml) were added Cs_2_CO_3_ (1.41 g, 4.33 mmol) and BnOH (4.53 ml, 43.5 mmol) and the mixture was stirred for 8 h at room temperature. The mixture was diluted H_2_O and organic materials were extracted with CHCl_3_. The combined extract was washed with brine, dried over Na_2_SO_4_, and concentrated *in vacuo*. The residue was purified by silica gel column chromatography (20 g). Elution with hexane-EtOAc (2:1) afforded the title compound (I) as a colorless solid (414 mg, 81%). The plate-like crystals of (I) were grown by slow cooling of a *t*-butyl­methyl­ether/hexane solution from *ca* 340 K to the room temperature. The specific rotation [α]_D_ of (I) at 295 K is -107° (*c* 1.33, EtOH).

### 1.2. Refinement

Crystal data, data collection and structure refinement details are summarized in Table 1. The absolute structure was assigned based on the known absolute configuration around the C6 atom, which originated from (+)-*D*-glucose. In the absence of significant anomalous scattering effects, Friedel pairs were averaged before the final refinement. There is an orientational disorder of the benzyl­oxy group, where the split phenyl group moieties lie on a common plane. The site occupation factor of the major part (O4A, C11A—C17A) was refined to 58.9 (9)%. All the H atoms were positioned geometrically, and refined as riding, with C—H = 0.93–0.98 Å and O—H = 0.82 Å, and with *U*_iso_(H) = 1.2*U*_eq_(C,O). The hy­droxy groups were allowed to rotate but not to tip.

### 2. Comment

Glycosidic bond formation on glycals such as (1a in Fig. 3) accompanied with the migration of double bond from C_1_—C_2_ to C_2_—C_3_ had been recognized as Ferrier reaction to give 2-eno­pyran­osides (2) (Ferrier & Prasad, 1969; Noshita *et al.*, 1995). Generally, α-glycosides predominate by the well known oxygen anomeric stabilizing effect, and the anomeric stereochemistry was elucidated by X-ray structure analysis of 2a (Wingert *et al.*, 1984). In contrast, Di Bussolo *et al.*(2002, 2004) have demonstrated unique β-selective approach, starting from glycals such as 3 with leaving group at C_4_—OH. By neighboring group participation with free C_3_—OH, *via* an epoxide inter­mediate (4), Ferrier-like rearrangement occurred to give 5. Their proposed mechanism is shown in Fig. 3. In this case, a hydrogen bonding between ep­oxy ring in 4 and nucleophile dominates the stereochemistry to β rather than α. We submitted 3,6-di-*O*-acetyl-*D*-glucal (1b), which is very easily available by an enzymatic regioselective acyl­ation of *D*-glucal (Calveras *et al.*, 2010), to Crotti's protocol. Introduction of methyl­sulfonyl group on free C_4_—OH (1c) and subsequent nuleophilic attack with benzyl alcohol provided the title compound, (I)(81%).

In the present study, the regio- and stereochemistry of (I) has been determined, although there is a complicated disorder. The benzyl­oxy group has two possible orientations, O4A/C11A—C17A and O4B/C11B—C17B, and their site occupation factors are 58.9 (9) and 41.1 (9)%, respectively. The C10—O4A and C10—O4B bond directions make an angle of 25.4 (7)°.

### Crystal data

**Table:**

C_13_H_16_O_4_	*F*(000) = 252.00
*M**_r_* = 236.26	*D*_x_ = 1.254 Mg m^−^^3^
Monoclinic, *P*2_1_	Melting point = 353–354 K
Hall symbol: P 2yb	Mo *K*α radiation, λ = 0.71069 Å
*a* = 19.500 (9) Å	Cell parameters from 25 reflections
*b* = 5.291 (2) Å	θ = 10.1–12.5°
*c* = 6.0809 (15) Å	µ = 0.09 mm^−^^1^
β = 94.27 (3)°	*T* = 292 K
*V* = 625.7 (4) Å^3^	Plate, colorless
*Z* = 2	0.60 × 0.40 × 0.20 mm

### Data collection

**Table:**

Rigaku AFC-7R diffractometer	*R*_int_ = 0.034
Radiation source: Rigaku rotating Mo anode	θ_max_ = 27.5°
Graphite plate monochromator	*h* = −9→25
ω–2θ scans	*k* = 0→6
1713 measured reflections	*l* = −7→7
1585 independent reflections	3 standard reflections every 150 reflections
786 reflections with *F*^2^ > 2σ(*F*^2^)	intensity decay: 0.8%

### Refinement

**Table:**

Refinement on *F*^2^	Secondary atom site location: difference Fourier map
Least-squares matrix: full	Hydrogen site location: inferred from neighbouring sites
*R*[*F*^2^ > 2σ(*F*^2^)] = 0.056	H-atom parameters constrained
*wR*(*F*^2^) = 0.255	*w* = 1/[σ^2^(*F*_o_^2^) + (0.1473*P*)^2^ + 0.0601*P*] where *P* = (*F*_o_^2^ + 2*F*_c_^2^)/3
*S* = 1.04	(Δ/σ)_max_ = 0.001
1579 reflections	Δρ_max_ = 0.16 e Å^−^^3^
193 parameters	Δρ_min_ = −0.17 e Å^−^^3^
33 restraints	Absolute structure: see text
Primary atom site location: structure-invariant direct methods	

### Special details

**Table:**

Experimental. Spectroscopic data: IR ν max: 3292, 2937, 2872, 1377, 1325, 1176, 1144, 1122, 1051, 970, 951, 876, 787, 744, 696 cm^-1^; ^1^H NMR (CDCl_3_): δ = 7.26–7.40 (m, 5H), 6.14 (ddd, *J* = 1.3, 4.6, 10.0 Hz, 1H), 5.89 (ddd, *J* = 1.0, 1.1, 10.0 Hz, 1H), 5.18 (ddd, *J* = 1.1, 1.3, 1.5 Hz, 1H), 4.92 (d, *J* = 11.8 Hz, 1H), 4.70 (d, *J* = 11.8 Hz, 1H), 4.02–4.09 (m, 1H), 3.97 (ddd, *J* = 5.9, 6.7, 12.0 Hz, 1H), 3.88 (ddd, *J* = 4.3, 7.3, 12.0 Hz, 1H), 3.79 (ddd, *J* = 3.1, 4.3, 6.7 Hz, 1H), 2.21 (dd, *J* = 5.9, 7.3 Hz, 1H), 1.97 (d, *J* = 10.6 Hz, 1H); ^13^C NMR (CDCl_3_): δ = 137.2, 130.9, 130.2, 128.4, 128.0, 127.9, 96.5, 75.1, 70.3, 62.9, 62.5.
Refinement. Refinement was performed using all reflections. The weighted *R*-factor (*wR*) and goodness of fit (*S*) are based on *F*^2^. *R*-factor (gt) are based on *F*. The threshold expression of *F*^2^ > 2σ(*F*^2^) is used only for calculating *R*-factor (gt).

### Fractional atomic coordinates and isotropic or equivalent isotropic displacement parameters (Å^2^)

**Table:**

	*x*	*y*	*z*	*U*_iso_*/*U*_eq_	Occ. (<1)
O1	0.3098 (2)	0.1914 (7)	0.7411 (7)	0.0920 (13)	
O2	0.4326 (4)	−0.2905 (9)	0.9178 (11)	0.152 (3)	
O3	0.4652 (2)	0.2679 (10)	0.7637 (8)	0.0988 (14)	
O4A	0.2323 (7)	0.485 (3)	0.5340 (18)	0.090 (4)	0.589 (9)
O4B	0.2402 (8)	0.476 (4)	0.638 (2)	0.068 (4)	0.411 (9)
C5	0.3777 (5)	−0.1093 (15)	0.9332 (13)	0.116 (3)	
C6	0.3606 (3)	0.0062 (11)	0.7108 (10)	0.0822 (15)	
C7	0.4216 (3)	0.1300 (14)	0.6082 (9)	0.0837 (16)	
C8	0.3960 (3)	0.2985 (17)	0.4253 (8)	0.0933 (19)	
C9	0.3317 (3)	0.3656 (15)	0.3939 (10)	0.096 (2)	
C10	0.2796 (3)	0.2768 (14)	0.5335 (12)	0.0946 (17)	
C11A	0.1683 (5)	0.409 (3)	0.605 (3)	0.097 (4)	0.589 (9)
C11B	0.1941 (7)	0.406 (4)	0.797 (4)	0.107 (6)	0.411 (9)
C12A	0.1400 (7)	0.603 (3)	0.754 (2)	0.078 (4)	0.589 (9)
C12B	0.1432 (11)	0.611 (4)	0.826 (4)	0.078 (4)	0.411 (9)
C13A	0.1743 (7)	0.635 (3)	0.960 (2)	0.108 (4)	0.589 (9)
C13B	0.1432 (12)	0.747 (5)	1.019 (4)	0.108 (4)	0.411 (9)
C14A	0.1520 (9)	0.811 (4)	1.107 (3)	0.153 (8)	0.589 (9)
C14B	0.0960 (16)	0.938 (7)	1.038 (5)	0.153 (8)	0.411 (9)
C15A	0.0936 (10)	0.947 (4)	1.041 (3)	0.143 (8)	0.589 (9)
C15B	0.0485 (15)	0.990 (5)	0.867 (4)	0.143 (8)	0.411 (9)
C16A	0.0592 (8)	0.927 (3)	0.837 (3)	0.109 (4)	0.589 (9)
C16B	0.0481 (10)	0.852 (4)	0.674 (3)	0.109 (4)	0.411 (9)
C17A	0.0842 (9)	0.750 (3)	0.696 (3)	0.095 (4)	0.589 (9)
C17B	0.0940 (15)	0.661 (5)	0.659 (5)	0.095 (4)	0.411 (9)
H2	0.4679	−0.2355	0.9822	0.1820*	
H3	0.4543	0.4176	0.7592	0.1185*	
H51	0.3919	0.0215	1.0387	0.1391*	
H52	0.3374	−0.1926	0.9836	0.1391*	
H6	0.3411	−0.1243	0.6103	0.0986*	
H7	0.4491	−0.0040	0.5463	0.1005*	
H8	0.4271	0.3589	0.3291	0.1120*	
H9	0.3188	0.4743	0.2779	0.1150*	
H10A	0.2558	0.1336	0.4596	0.1135*	0.589 (9)
H10B	0.2496	0.1490	0.4601	0.1135*	0.411 (9)
H11A	0.1737	0.2499	0.6832	0.1165*	0.589 (9)
H11B	0.1361	0.3836	0.4779	0.1165*	0.589 (9)
H11C	0.2197	0.3725	0.9365	0.1280*	0.411 (9)
H11D	0.1701	0.2528	0.7494	0.1280*	0.411 (9)
H13A	0.2127	0.5360	1.0006	0.1298*	0.589 (9)
H13B	0.1750	0.7105	1.1365	0.1298*	0.411 (9)
H14A	0.1753	0.8367	1.2442	0.1836*	0.589 (9)
H14B	0.0965	1.0323	1.1672	0.1836*	0.411 (9)
H15A	0.0767	1.0598	1.1409	0.1720*	0.589 (9)
H15B	0.0164	1.1182	0.8812	0.1720*	0.411 (9)
H16A	0.0211	1.0264	0.7962	0.1307*	0.589 (9)
H16B	0.0168	0.8893	0.5555	0.1307*	0.411 (9)
H17A	0.0621	0.7311	0.5564	0.1141*	0.589 (9)
H17B	0.0920	0.5625	0.5324	0.1141*	0.411 (9)

### Atomic displacement parameters (Å^2^)

**Table:**

	*U*^11^	*U*^22^	*U*^33^	*U*^12^	*U*^13^	*U*^23^
O1	0.089 (3)	0.071 (3)	0.121 (3)	0.007 (2)	0.040 (3)	−0.013 (3)
O2	0.198 (6)	0.072 (3)	0.169 (5)	0.023 (4)	−0.094 (5)	−0.033 (3)
O3	0.083 (3)	0.091 (3)	0.117 (3)	0.003 (3)	−0.029 (2)	−0.023 (3)
O4A	0.079 (6)	0.079 (5)	0.111 (8)	0.007 (5)	0.009 (7)	0.007 (8)
O4B	0.051 (6)	0.067 (6)	0.087 (9)	0.012 (5)	0.010 (7)	0.010 (9)
C5	0.132 (6)	0.088 (5)	0.128 (5)	0.001 (5)	0.010 (5)	0.019 (5)
C6	0.089 (4)	0.060 (3)	0.097 (4)	0.007 (3)	0.003 (3)	−0.022 (3)
C7	0.076 (3)	0.093 (4)	0.082 (3)	0.020 (3)	−0.000 (3)	−0.032 (3)
C8	0.080 (4)	0.128 (6)	0.073 (3)	0.004 (4)	0.012 (3)	−0.012 (4)
C9	0.086 (4)	0.118 (6)	0.083 (3)	0.005 (4)	−0.001 (3)	−0.008 (4)
C10	0.067 (3)	0.075 (4)	0.141 (5)	0.010 (4)	0.005 (4)	−0.015 (4)
C11A	0.057 (5)	0.094 (8)	0.141 (9)	−0.013 (6)	0.016 (6)	−0.026 (8)
C11B	0.060 (8)	0.116 (13)	0.146 (15)	0.024 (9)	0.023 (9)	0.042 (13)
C12A	0.062 (4)	0.084 (4)	0.088 (10)	0.003 (4)	0.002 (6)	0.018 (6)
C12B	0.062 (4)	0.084 (4)	0.088 (10)	0.003 (4)	0.002 (6)	0.018 (6)
C13A	0.107 (9)	0.109 (10)	0.106 (8)	0.002 (7)	−0.009 (7)	−0.000 (8)
C13B	0.107 (9)	0.109 (10)	0.106 (8)	0.002 (7)	−0.009 (7)	−0.000 (8)
C14A	0.159 (14)	0.19 (2)	0.107 (10)	−0.077 (16)	0.007 (9)	−0.040 (11)
C14B	0.159 (14)	0.19 (2)	0.107 (10)	−0.077 (16)	0.007 (9)	−0.040 (11)
C15A	0.169 (16)	0.104 (10)	0.171 (17)	0.018 (11)	0.109 (13)	0.011 (11)
C15B	0.169 (16)	0.104 (10)	0.171 (17)	0.018 (11)	0.109 (13)	0.011 (11)
C16A	0.096 (7)	0.100 (9)	0.134 (11)	0.022 (7)	0.031 (8)	−0.012 (7)
C16B	0.096 (7)	0.100 (9)	0.134 (11)	0.022 (7)	0.031 (8)	−0.012 (7)
C17A	0.076 (7)	0.087 (13)	0.121 (7)	0.032 (8)	0.004 (6)	0.004 (8)
C17B	0.076 (7)	0.087 (13)	0.121 (7)	0.032 (8)	0.004 (6)	0.004 (8)

### Geometric parameters (Å, º)

**Table:**

O1—C6	1.416 (8)	C16B—C17B	1.36 (4)
O1—C10	1.426 (8)	O2—H2	0.820
O2—C5	1.445 (11)	O3—H3	0.820
O3—C7	1.424 (8)	C5—H51	0.970
O4A—C10	1.438 (17)	C5—H52	0.970
O4A—C11A	1.409 (18)	C6—H6	0.980
O4B—C10	1.477 (19)	C7—H7	0.980
O4B—C11B	1.42 (3)	C8—H8	0.930
C5—C6	1.499 (10)	C9—H9	0.930
C6—C7	1.531 (9)	C10—H10A	0.980
C7—C8	1.483 (9)	C10—H10B	0.980
C8—C9	1.304 (9)	C11A—H11A	0.970
C9—C10	1.449 (9)	C11A—H11B	0.970
C11A—C12A	1.499 (19)	C11B—H11C	0.970
C11B—C12B	1.49 (3)	C11B—H11D	0.970
C12A—C13A	1.388 (18)	C13A—H13A	0.930
C12A—C17A	1.36 (3)	C13B—H13B	0.930
C12B—C13B	1.38 (3)	C14A—H14A	0.930
C12B—C17B	1.37 (4)	C14B—H14B	0.930
C13A—C14A	1.38 (3)	C15A—H15A	0.930
C13B—C14B	1.38 (4)	C15B—H15B	0.930
C14A—C15A	1.38 (3)	C16A—H16A	0.930
C14B—C15B	1.37 (4)	C16B—H16B	0.930
C15A—C16A	1.37 (3)	C17A—H17A	0.930
C15B—C16B	1.39 (3)	C17B—H17B	0.930
C16A—C17A	1.38 (3)		
			
C6—O1—C10	110.5 (5)	C7—C6—H6	108.989
C10—O4A—C11A	111.4 (12)	O3—C7—H7	108.061
C10—O4B—C11B	118.8 (16)	C6—C7—H7	108.071
O2—C5—C6	109.1 (7)	C8—C7—H7	108.069
O1—C6—C5	106.0 (6)	C7—C8—H8	118.624
O1—C6—C7	109.3 (5)	C9—C8—H8	118.631
C5—C6—C7	114.5 (6)	C8—C9—H9	118.863
O3—C7—C6	113.1 (5)	C10—C9—H9	118.877
O3—C7—C8	109.9 (6)	O1—C10—H10A	108.127
C6—C7—C8	109.5 (5)	O1—C10—H10B	112.184
C7—C8—C9	122.7 (6)	O4A—C10—H10A	108.127
C8—C9—C10	122.3 (6)	O4B—C10—H10B	112.183
O1—C10—O4A	117.6 (7)	C9—C10—H10A	108.128
O1—C10—O4B	92.3 (7)	C9—C10—H10B	112.184
O1—C10—C9	111.1 (5)	O4A—C11A—H11A	109.342
O4A—C10—C9	103.3 (8)	O4A—C11A—H11B	109.349
O4B—C10—C9	115.4 (9)	C12A—C11A—H11A	109.342
O4A—C11A—C12A	111.4 (11)	C12A—C11A—H11B	109.335
O4B—C11B—C12B	110.7 (18)	H11A—C11A—H11B	107.980
C11A—C12A—C13A	117.0 (12)	O4B—C11B—H11C	109.503
C11A—C12A—C17A	123.9 (12)	O4B—C11B—H11D	109.498
C13A—C12A—C17A	119.1 (14)	C12B—C11B—H11C	109.496
C11B—C12B—C13B	121.7 (19)	C12B—C11B—H11D	109.495
C11B—C12B—C17B	119 (2)	H11C—C11B—H11D	108.069
C13B—C12B—C17B	119 (2)	C12A—C13A—H13A	119.650
C12A—C13A—C14A	120.7 (13)	C14A—C13A—H13A	119.655
C12B—C13B—C14B	120 (2)	C12B—C13B—H13B	120.088
C13A—C14A—C15A	117.1 (15)	C14B—C13B—H13B	120.082
C13B—C14B—C15B	120 (3)	C13A—C14A—H14A	121.432
C14A—C15A—C16A	124.1 (18)	C15A—C14A—H14A	121.427
C14B—C15B—C16B	120 (3)	C13B—C14B—H14B	119.853
C15A—C16A—C17A	116.3 (15)	C15B—C14B—H14B	119.850
C15B—C16B—C17B	119 (2)	C14A—C15A—H15A	117.945
C12A—C17A—C16A	122.6 (15)	C16A—C15A—H15A	117.953
C12B—C17B—C16B	122 (3)	C14B—C15B—H15B	120.031
C5—O2—H2	109.474	C16B—C15B—H15B	120.027
C7—O3—H3	109.475	C15A—C16A—H16A	121.839
O2—C5—H51	109.847	C17A—C16A—H16A	121.842
O2—C5—H52	109.853	C15B—C16B—H16B	120.433
C6—C5—H51	109.854	C17B—C16B—H16B	120.424
C6—C5—H52	109.864	C12A—C17A—H17A	118.712
H51—C5—H52	108.286	C16A—C17A—H17A	118.718
O1—C6—H6	108.978	C12B—C17B—H17B	119.115
C5—C6—H6	108.981	C16B—C17B—H17B	119.099
			
C6—O1—C10—O4A	−173.3 (5)	C8—C9—C10—O4A	147.8 (7)
C6—O1—C10—O4B	−173.0 (4)	C8—C9—C10—O4B	124.1 (7)
C6—O1—C10—C9	−54.6 (6)	O4A—C11A—C12A—C13A	−68.8 (15)
C10—O1—C6—C5	−168.0 (4)	O4A—C11A—C12A—C17A	109.9 (13)
C10—O1—C6—C7	68.1 (5)	O4B—C11B—C12B—C13B	−111.9 (19)
C10—O4A—C11A—C12A	139.3 (9)	O4B—C11B—C12B—C17B	70 (2)
C11A—O4A—C10—O1	−74.9 (11)	C11A—C12A—C13A—C14A	179.4 (11)
C11A—O4A—C10—O4B	−75.6 (14)	C11A—C12A—C17A—C16A	179.8 (12)
C11A—O4A—C10—C9	162.3 (9)	C13A—C12A—C17A—C16A	−2 (3)
C10—O4B—C11B—C12B	−160.8 (10)	C17A—C12A—C13A—C14A	1 (2)
C11B—O4B—C10—O1	−58.0 (13)	C11B—C12B—C13B—C14B	178.8 (17)
C11B—O4B—C10—O4A	121 (3)	C11B—C12B—C17B—C16B	−177.4 (19)
C11B—O4B—C10—C9	−172.7 (11)	C13B—C12B—C17B—C16B	4 (4)
O2—C5—C6—O1	−176.5 (5)	C17B—C12B—C13B—C14B	−3 (4)
O2—C5—C6—C7	−56.0 (7)	C12A—C13A—C14A—C15A	2 (3)
O1—C6—C7—O3	77.9 (6)	C12B—C13B—C14B—C15B	1 (4)
O1—C6—C7—C8	−45.0 (6)	C13A—C14A—C15A—C16A	−3 (3)
C5—C6—C7—O3	−40.7 (8)	C13B—C14B—C15B—C16B	−1 (5)
C5—C6—C7—C8	−163.7 (5)	C14A—C15A—C16A—C17A	3 (3)
O3—C7—C8—C9	−111.7 (7)	C14B—C15B—C16B—C17B	2 (4)
C6—C7—C8—C9	13.0 (9)	C15A—C16A—C17A—C12A	−0 (3)
C7—C8—C9—C10	−1.1 (11)	C15B—C16B—C17B—C12B	−4 (4)
C8—C9—C10—O1	20.8 (10)		

### Hydrogen-bond geometry (Å, º)

**Table:**

*D*—H···*A*	*D*—H	H···*A*	*D*···*A*	*D*—H···*A*
O2—H2···O3^i^	0.82	1.95	2.692 (8)	151
O3—H3···O2^ii^	0.82	1.89	2.614 (8)	147

Symmetry codes: (i) −*x*+1, *y*−1/2, −*z*+2; (ii) *x*, *y*+1, *z*.
